# Metaproteomic analysis of human sonicate fluid highlights neutrophil extracellular trap formation associated with periprosthetic joint infection

**DOI:** 10.1128/spectrum.00665-26

**Published:** 2026-10-13

**Authors:** Elijah Garcia, Kathleen O'Connor, Christina Koscianski, Judith Alvarez Otero, Keara O’Donnell, Kiran K. Mangalaparthi, Nicholas B. Larson, Akhilesh Pandey, David Holmes, Robin Patel

**Affiliations:** 1 Graduate School of Biomedical Sciences, Mayo Clinic, Rochester, Minnesota, USA; 2 Alix School of Medicine, Mayo Clinic, Rochester, Minnesota, USA; 3 Division of Clinical Microbiology, Department of Laboratory Medicine and Pathology, Mayo Clinic 195112, Rochester, Minnesota, USA; 4 Department of Laboratory Medicine and Pathology, Mayo Clinic 195112, Rochester, Minnesota, USA; 5 Department of Quantitative Health Sciences, Mayo Clinic 6915, Rochester, Minnesota, USA; 6 Physiology and Biomedical Engineering, Mayo Clinic 6915, Rochester, Minnesota, USA; 7 Division of Public Health, Infectious Diseases, and Occupational Medicine, Department of Medicine, Mayo Clinic 6915, Rochester, Minnesota, USA; LSU Health Shreveport, Shreveport, Louisiana, USA

**Keywords:** proteomics, periprosthetic joint infection, neutrophil extracellular traps

## Abstract

Periprosthetic joint infection (PJI) is a devastating complication of total joint arthroplasty that can be challenging to diagnose and treat. Neutrophils are a prominent feature in PJI, with neutrophil extracellular traps (NETs) known to be present; little is known, however, about the physiology of PJI-associated NETs. Here, the proteomes of PJI and non-infectious arthroplasty failure (NIAF), analyzed using liquid chromatography tandem mass-spectrometry (LC-MS/MS) and proximity extension assay (PEA)-based profiling of fluids derived from sonication of arthroplasty components removed from patients who had undergone revision arthroplasty, were compared, with a focus on NET formation. In addition, *in vitro* effects of synovial fluid components on *Staphylococcus aureus-*induced NET formation were characterized. LC-MS/MS (PJI *n* = 85, NIAF *n* = 26) and PEA (PJI *n* = 95, NIAF *n* = 39) showed NET formation and neutrophil degranulation to be associated with PJI, with the complement cascade and platelet activation pathways identified as potential contributors. *In vitro* studies showed delayed NET formation in artificial synovial fluid compared to standard cell culture media conventionally used to evaluate NETs. Together, these results demonstrate the presence of NETs in PJI, identify potential pathways involved in PJI-associated NETosis, and point to an influence of synovial components on NET formation during PJI.

**IMPORTANCE:**

Periprosthetic joint infection (PJI) is a major complication of joint replacement, placing a significant burden on patients and the healthcare system. Our research reveals that neutrophils and their antimicrobial “traps” are dominant features of the immune response at the infection joint site. Importantly, we found that the unique environment of the joint delays the formation of these traps, potentially contributing to the pathogenesis of PJI. By establishing a new experimental platform tailored for the infected joint, this work provides a foundational platform for probing these mechanisms and supports the development of improved diagnostic and therapeutic strategies.

## INTRODUCTION

Periprosthetic joint infection (PJI) is the most common and difficult to treat cause of total joint arthroplasty (TJA) failure (1). Relatively steady PJI rates combined with increasing TJA volumes are projected to lead to an annual hospital cost of $1.85 billion for PJI in the United States by 2030 (2). Beyond economic burden, the 10-year cumulative mortality is 44% and 36% for those with PJI affecting the hip and knee, respectively (3, 4). Distinguishing PJI from non-infectious arthroplasty failure (NIAF) can be challenging in some cases, as both conditions may present similarily (5). Prompt and accurate diagnosis of PJI informs correct selection of surgical management and antimicrobial therapy, but despite multiple iterations of diagnostic criteria for PJI (6, 7), diagnosing PJI accurately can be challenging. Even if PJI is accurately diagnosed, it can be difficult to treat, with infections recurring despite aggressive antibiotic treatment and surgery (8, 9). Finally, prevention strategies, although effective (10), are insufficient and have not fundamentally changed PJI rates in recent years. To improve and fine-tune PJI management, including diagnosis, treatment, and prevention, a better understanding of the physiology of this disease is needed.

Neutrophils, a heterogeneous population of innate immune cells that act as a first line of defense against infection, are elevated in PJI (11); their presence is routinely assessed in synovial fluid and periprosthetic tissues for diagnosis (5, 11–16). Considered to be associated with acute infection, their persistent abundance throughout the course of PJI is somewhat paradoxical and may provide a potential clue as to its pathogenesis (11, 17). Recent studies have shown neutrophil extracellular traps (NETs) to be more abundant in PJI than NIAF (18–20). Described *in vitro* two decades ago, NETs are web-like structures studded with antimicrobial proteins that ensnare and kill microbes (21). As with neutrophils themselves, the presence of NETs in PJI suggests that NETs may not be performing as intended, an understanding of which could help explain the pathogenesis of PJI and point to diagnostic, therapeutic, and prevention strategies. Importantly, beyond being present, the physiology and role of NETs in PJI is, for the most part, unexplored.

Understanding the physiology of NETosis in PJI will provide insight as to why neutrophils and associated NETs do not clear infections. It is hypothesized that pathways activated during PJI NETosis may be unique compared to other infection-types, as stimulation source and local host physiology result in a range of NET forms. Indeed, under pathologic conditions, neutrophils have distinct transcriptomes depending on the environment (e.g., synovial fluid *versus* blood) (22) and form NETs with different morphologies based on organ type (23). Additionally, patients with lupus or diabetes have NETs with distinct proteomes compared to healthy individuals (24, 25), and neutrophils from patients with rheumatoid arthritis undergo distinct NET signaling pathways (26). While associated with beneficial functions (i.e., antimicrobial activity), NETs can lead to negative effects on the host (e.g., autoimmunity) (27, 28). NETosis can be broadly categorized as undergoing lytic and non-lytic (“vital”) processes (29), with molecular mechanisms underpinning each subtype incompletely defined. More complex categorization—such as whether NETs are engaging NET pathways that are helpful or hurtful to the host in PJI—is unexplored.

In this study, proteomes of fluids derived from sonication of arthroplasty components removed from patients who had undergone revision knee arthroplasty for PJI or NIAF (30) were analyzed using liquid chromatography tandem mass-spectrometry (LC-MS/MS) and proximity extension assay (PEA). Using the PEA system, matched antibody pairs bind target proteins to trigger extension of conjugated DNA oligos into a unique barcode. This translates protein concentration into a DNA-based readout that can be assessed using next-generation sequencing, enabling precise quantification of hundreds of biomarkers. The two proteomic techniques have demonstrated complementary advantages in deciphering complex proteomes of biological fluids (31–33), including plasma (34, 35). Pathways related to NET formation and neutrophil degranulation were assessed in PJI, and specific pathways involved were defined. Further, to determine how the unique microenvironment of the periprosthetic joint space impacts the dynamics of NETosis, an *in vitro* model of PJI NET formation involving *Staphylococcus aureus* bead biofilms in artificial synovial fluid was used to assess NETosis. Findings presented show NETosis to be abundant in PJI and a key feature of PJI pathophysiology, identify potential pathways involved in PJI-associated NETosis, and point to a possible influence of synovial components on NET formation during PJI.

## MATERIALS AND METHODS

### Proteomic studies

#### Clinical samples and implant sonication

Sonicate fluids were collected between October 2007 and March 2022 from 135 total knee arthroplasty revisions under Mayo Clinic (Rochester, MN) Institutional Review Board 09-000808 and were prepared as previously described (30). Briefly, explanted arthroplasty components were transferred into sterile containers in the operating room. In the clinical microbiology laboratory, each container received 400 mL of sterile Ringer’s solution and was subjected to the following: 30 s of vortexing, 5 min of sonication in an ultrasound bath at 40 kHz, and a final 30 s vortex. Prior to concentration, 3 mL of sonicate fluid was aliquoted and stored at −80°C for future PEA analysis. The remaining sonicate fluid was centrifuged at 3,150 × *g* for 5 min, yielding an 8 mL pellet, which was preserved at −80°C for future LC-MS/MS analysis. A diagnosis of PJI was made if at least one of the following was present: a sinus tract communicating with the prosthesis; visibly purulent synovial fluid or visible purulence surrounding the prosthesis; acute inflammation on histopathological examination of permanent tissue sections as determined by a pathologist; or two or more intraoperative tissue cultures or a combination of preoperative aspiration culture and an intraoperative culture yielding the same organism (36). NIAF was defined as failure of a prosthesis in the absence of these criteria. Patients diagnosed with PJI were preferentially selected for further sonicate fluid analysis based on sonicate fluid culture positivity with >51 colony-forming units (CFU)/mL of a bacterial isolate. Of the 95 PJI samples, 30 were infected with *Staphylococcus epidermidis*, 19 *Staphylococcus aureus*, 9 *Enterococcus faecalis*, 6 *Staphylococcus lugdunensis*, 4 *Streptococcus agalactiae*, 3 *Pseudomonas aeruginosa*, 3 *Staphylococcus caprae,* 2 *Cutibacterium acnes*, 2 *Staphylococcus capitis*, 1 *Candida albicans*, and 16 other unique bacterial species. The uninfected samples were categorized into three subgroups: 17 instability, 14 aseptic loosening, and nine osteolysis. One hundred thirty-five unconcentrated sonicate fluid samples were analyzed by PEA. One sample did not meet PEA quality-control criteria and was excluded from PEA analysis, but was analyzed by LC-MS/MS. One hundred eleven concentrated samples from a subset of sonicate fluids tested by PEA underwent analysis by LC-MS/MS.

#### Sonicate fluid preparation for LC-MS/MS

Sonicate fluids were processed for LC-MS/MS analysis as previously described (33). Briefly, 300 µL of sonicate fluid was supplemented with 20% sodium dodecyl sulfate (SDS) to achieve a 1% final concentration, along with 1× HALT protease and phosphatase inhibitors (Thermo Fisher Scientific, Waltham, MA). Following a 20 s vortex, the mixture underwent probe sonication at 15% amplitude for 30 s on ice. Debris was removed via centrifugation at 15,000 × *g* for 5 min. The resulting supernatant was collected, and protein concentrations were determined using a BCA assay (Thermo Fisher Scientific). A 100 µg protein aliquot was vacuum-dried and reconstituted in a buffer of 5% SDS and 50 mM triethylammonium bicarbonate (TEABC, pH 8.5). Proteins were reduced with 5 mM dithiothreitol for 45 min and alkylated with 20 mM iodoacetamide for 30 min. Further processing was performed in S-Trap 96-well Mini Plates (Protifi, Fairport, NY) according to the manufacturer’s recommendations: acidified lysates were diluted in 90% methanol, supplemented with 100 mM TEABC, loaded onto the plate, and washed twice after centrifugation at 2,000 × *g* for 2 min. After overnight trypsinization at 37°C, peptides were eluted, dried using SpeedVac concentrator, and preserved at −80°C for subsequent LC-MS/MS analysis.

#### LC-MS/MS analysis

LC-MS/MS analysis was conducted using a timsTOF HT mass spectrometer (Bruker Daltonics, Bremen, Germany) coupled to an UltiMate 3000 RSLCnano system (Thermo Fisher Scientific). Peptides were resuspended in 0.2% formic acid, 0.1% trifluoroacetic acid, and 0.0001% Zwittergent 3-16 and loaded onto an EXP2 stem trap column (Optimize Technologies, Oregon City, OR), with subsequent separation on a 25 cm PepSep Extreme C_18_ column (25 cm × 150 μm × 1.5 µm C_18_ column, Bruker). Peptide separation was performed using a gradient of 2% mobile phase A (0.1% formic acid in water) to 40% mobile phase B (80% acetonitrile, 0.1% formic acid) in 45 min. This was followed by a 5 min ramp to 90% mobile phase B solution, followed by re-equilibration to the starting gradient conditions. Eluted peptides were analyzed using data-dependent acquisition with parallel accumulation-serial fragmentation mode in the mass spectrometer. A 100% duty cycle was maintained with an accumulation and ramp time of 100 ms over an inverse ion mobility range (1/*K*_0_) range of 0.7 to 1.43 in 100 ms. Mass range for MS and MS/MS analysis was specified as 100–1,700 m/z, and precursors were fragmented using a collision energy setting that increased linearly from 20 eV at 1/*K*_0_ = 0.6 Vscm^−2^ to 59 eV at 1/*K*_0_ = 1.6 Vs/cm^2^. For protein identification and quantification, FragPipe software (version 21.1) was utilized. MS/MS spectra were queried against the fragment ion index derived from the human UniProt protein database and contaminant proteins. Default database-searching parameters were used, including precursor and fragment ion tolerances of 20 ppm, trypsin cleavage specificity set to allow two missed cleavages, a peptide length of 7–50 amino acids, and a mass range of 500 to 5000 Daltons. Cysteine carbamidomethylation was set as a specific modification, while methionine oxidation and N-terminal acetylation were set as variable modifications. Results were rescored with the MSBooster algorithm and validated with Percolator. The false discovery rate (FDR) was controlled by the Philosopher tool (version 5.1) with a sequential FDR approach to 1% protein-level FDR. Protein quantitation and normalization were performed using IonQuant (version 1.10.12) through the MaxLFQ method. For data analysis, LC-MS/MS data with 0 values were replaced with the “minprob” function with a *q* = 0.01 from the DEP R package (version 1.30.0), as described (37); quantile normalization was performed prior to log_2_ transformation.

#### Protein analysis via PEA

Protein profiling via PEA was performed on the Olink Explore 3072 platform (Olink Proteomics, Uppsala, Sweden), according to the manufacturer’s instructions. Protein detection began with the binding of two antibodies tagged with unique oligonucleotides to a protein. Complementarity of the oligos and subsequent hybridization was used to amplify the signals through the generation of a DNA library, which was sequenced on a NovaSeq 6000 SP flow cell (Illumina, San Diego, CA). Following sequencing, raw data were demultiplexed using the Olink “bcl2counts” script, and quality control of read counts was evaluated using NPX Explore software (Olink Proteomics). Resulting data were exported for further manual normalization based on individual sample protein concentrations. Prior to data analysis, data were normalized, as previously described (33). Briefly, protein expression values were adjusted for concentration using raw read counts, internal spike-in controls (extension controls), and measured protein concentrations. Raw counts were first normalized by dividing by the corresponding extension control count to obtain preliminary normalized expression values. A relative concentration factor (“c”) was then calculated for each sample as the ratio of its total protein concentration to the overall mean protein concentration across all samples. The preliminary normalized expression value was multiplied by its respective “c” factor to produce concentration-adjusted abundance values. Finally, quantile normalization was applied using the “normalize.quantiles” function from the preprocessCore package (version 1.70.0) (38), and the results were log_2_-transformed to yield the final normalized protein expression data set.

#### Univariate analysis

To identify differentially abundant proteins (DAPs) between PJI and NIAF, as well as host protein signatures associated with specific PJI microbial etiologies compared to NIAF, the base R (version 4.5.0) stats package was utilized to compare group means of each protein using two-tailed “t.test” and “p.adjust” to adjust *P-*values for multiple comparisons through the Benjamini–Hochberg (BH) FDR procedure. DAPs were considered significant if they had an adjusted *P*-value < 0.05. For microorganism-specific analyses, PJI caused by bacterial species with ≥5 biological replicates was included, including PJI caused by *S. epidermidis* (LC-MS/MS *n* = 26, PEA *n* = 30), *S. aureus* (LC-MS/MS *n* = 18, PEA *n* = 19), *E. faecalis* (LC-MS/MS and PEA *n* = 9), and *S. lugdunensis* (LC-MS/MS *n* = 5, PEA *n* = 6). The cutpointr R package (version 1.2.1) (39) was used to estimate single protein thresholds for discriminating PJI and NIAF. Area under the curve (AUC) values were calculated from resulting receiver operating characteristic curves. The top 30 protein predictors based on AUC were selected for heatmap visualization via the ComplexHeatmap R package (version 2.24.1) (40, 41). K-means clustering was applied to both rows and columns using Euclidean as the distance metric. Protein values were not further scaled prior to clustering and represent normalized abundances from data processing.

#### Enrichment and pathway analysis

To map DAPs to relevant pathways, overrepresentation analysis was performed using the clusterProfiler R package (version 4.16.0) (42). Pathway databases used included the Kyoto Encyclopedia of Genes and Genomes (KEGG) (43), Reactome (44), Gene Ontology (45), and cell markers from clusterProfiler, as shown in Table S1. Default enrichment parameters were used for each pathway database query; multiple comparisons were adjusted through the BH procedure, with an adjusted *P-*value cutoff of < 0.05, and a range of gene size for each term set at 10–500 genes. The org.Hs.eg.db database of human proteins (version 3.21.0) (46) was used, and background protein sets (“universes”) were defined as all 5,793 or 2,888 proteins measured by LC-MS/MS or PEA, respectively. Protein lists and universes used are shown in supplementary material (Tables S2 to S7). Gene-concept maps were produced from the “cnetplot” function of enrichplot R package (version 1.28.2) (47), with a custom R script adjusting the “ggplot” object. Consistently identified significantly elevated proteins in PJI were mapped to upstream mechanisms of NET formation (“hsa04613”) through the Pathview R package (version 1.48.0) (48). Log_2_ fold changes from LC-MS/MS and PEA data sets were individually scaled from −2 to 2 with the “‘scale” function of the base R stats package for integration into the same pathway image under a common scale.

#### Multivariate analysis

Multivariate protein profile differences between PJI and NIAF were assessed by applying sparse partial least squares differential analysis (sPLS-DA) using the mixOmics R package (version 6.32.0) (49). Numbers of components and features per component were determined through the “tune.splsda” function; if tuning selected a ncomp of 1, a ncomp of 2 was selected as that is the minimum requirement of the program. Validation of the sPLS-DA model was performed with the “perf” function, using 5-fold cross-validation with 50 iterations (M-fold).

### Benchmarking of sonicate fluid proteome against a published synovial fluid proteome

To assess alignment between sonicate and synovial fluid, a published LC-MS/MS data set of PJI (*n* = 8) and NIAF (*n* = 13) synovial fluids was used as a biological benchmark for sonicate fluid findings (50). The univariate analysis and overrepresentation analysis were performed as described for the sonicate fluid data set. A threshold of *P*-value of < 0.05, without adjustment for multiple comparisons for DAPs, was used for pathway analysis, as no proteins had an adjusted *P*-value < 0.05. Prior to multivariate analysis, sonicate and synovial fluid data sets were individually scaled from −2 to 2 using the “scale” function to facilitate cross-data set comparison for the sPLS-DA pipeline.

### *In vitro* studies of synovial fluid effects on *S. aureus* biofilm-induced NET formation

#### Neutrophil preparation

Neutrophils were isolated from nine healthy human donors, under Mayo Clinic IRB 24-006768. Whole blood was collected through venous puncture into 6 mL BD Vacutainer EDTA tubes to prevent coagulation and used immediately. Neutrophils were isolated as described by Oh et al. (51). Briefly, whole blood was slowly pipetted onto Lympholyte-Poly (Cedarlane Laboratories, Ontario, Canada) and centrifuged at 500 × *g* for 35 min. Plasma and monocyte layers were removed, and the neutrophil and isolation medium layers were isolated, avoiding red blood cells to the extent possible. Remaining red blood cells were lysed with Red Cell Lysis buffer (Roche, Basel, Switzerland), and neutrophils were washed with Hank’s Balanced Salt Solution without calcium or magnesium. To mimic synovial fluid conditions, artificial synovial fluid (aSF), referred to as “SSF2” by De Bleeckere et al., was prepared as previously described (52) with the final composition shown in Table S10. Neutrophils were resuspended in aSF without fibrinogen prior to counting with a hemocytometer.

#### *S. aureus* bead biofilm formation

*S. aureus* IDRL-12882, a clinical isolate obtained from an total knee arthroplasty revision for PJI, was retrieved from freezer stock, streaked onto blood agar, and incubated overnight at 37°C. Colonies were transferred to 14 mL Falcon tubes with 3 mL tryptic soy broth, and 5 sterile 4 mm glass beads (MilliporeSigma, Burlington, MA) and grown to stationary phase (24 h, 37°C). Beads were washed thrice by vortexing for 30 s in 5 mL phosphate-buffered saline (PBS) solution without calcium or magnesium (Cytiva, Marlborough, MA). For biofilm quantification, cultures were grown in triplicate, and a single bead from each culture was sonicated for 5 min in 5 mL of PBS prior to serial dilution and plating 100 µL onto blood agar plates. Plates were incubated at 37°C overnight prior to colony counting.

#### Visualization and quantification of NETs stimulated by *S. aureus* biofilms using time-lapse fluorescence microscopy

In a 24-well plate, 2 × 10^5^ neutrophils were seeded with biofilm-coated or sterile glass beads in either aSF or Roswell Park Memorial Institute (RPMI-1640; Sigma Aldrich, Burlington, MA) medium. During model development, 100 μL of filter-sterilized supernatant from 24 h cultures, grown stationarily in test tubes, was assessed as an alternative method for induction of NETs. To assess the impact of hyaluronic acid and bovine serum albumin (BSA) on *S. aureus* induction of NETosis *in vitro*, six media compositions were tested. aSF was created as previously described (52), with the exception that bovine fibrinogen was used in place of human fibrinogen. Additionally, two variants were created, with one without added BSA and the other without added sodium hyaluronate. Three mixtures of RPMI-1640 medium were prepared; all used RPMI-1640 without phenol red and were supplemented with 50 mM HEPES and 0.05% human serum albumin. One mixture was made with no other additions, one with BSA (2.5 g/1 L) added to a final concentration of 2.5% BSA, and one with sodium hyaluronate added to a final concentration of 0.3%. Membrane-permeable NUCLEAR-ID red (Enzo Life Sciences, Farmingdale, NY) and membrane-impermeable Sytox Green (NucGreen Dead 488 ReadyProbe Reagent, Invitrogen, Thermo Fisher Scientific, Waltham, MA) dyes were added to each well, and cells were incubated at 37°C with time-lapse images taken every 1 h for 24 h with a Keyence BZ-X800 (Keyence, Osaka, Japan) at 10× magnification.

Time-lapse images were analyzed using a bespoke macro for Fiji/ImageJ (version 2.14.0) (53). The analysis method for tracking and counting neutrophils and NETs separated images into two color channels, with red designating live neutrophils and green designating NETs. Images for each channel were adjusted by applying the commands, “Gaussian Blur,” “Enhance Contrast,” and “Subtract Background,” to ensure cells were separated from background. Renyi’s entropy was applied to establish a binary mask to separate cells from background for counting (54). Cells were counted using the command “Analyze Particles,” with a minimum size threshold of ≥10 pixels for NETs or neutrophils, established by running the command “Set Measurements” and toggling the “Median Parameter” on initial analysis; at an image scale of 0.75 µm/pixel, this corresponded to a size of 7.5 µm, about half the size of an average neutrophil (55). This process was repeated at every time interval, and the resulting data were exported as .csv files for analysis. Data were imported into R and normalized by dividing the percentage of NETs by the total cell number, resulting in a NETs ratio. For each experimental condition, replicate wells were averaged at each time point. Summary statistics were calculated as mean, with error bars representing standard error of mean (SEM).

### Figure generation

Figures were generated using GraphPad Prism (Graphpad Software, Boston, MA) and R Studio (version 2025.05.1 + 513). Volcano and dot plots were generated from using the “ggplot” and “geom_point” functions from the ggplot2 R package (version 3.5.2) (56).

## RESULTS

### Subject demographics

One hundred thirty-four subjects were enrolled in the arthroplasty failure study, 95 with PJI and 39 NIAF. Sonicate fluids from all 134 were analyzed by PEA, with a subset of 111 (PJI *n* = 85, NIAF *n* = 26) assessed by LC-MS/MS. Demographic and preoperative laboratory values were similar between the groups (Table 1), except that, as expected, those with PJI had a higher median erythrocyte sedimentation rate (ESR), C-reactive protein (CRP), synovial fluid total nucleated cell count, and synovial polymorphonuclear cell percentage values compared with those with NIAF; those with PJI were also older than those with NIAF.

**TABLE 1:** Patient demographics^*c*^

Characteristic	Periprosthetic joint infection (*n* = 95)	Non-infectious arthroplasty failure (*n* = 40)	*P*-value
Age (years), median (IQR)	66.0 (13.5)	63.5 (12.2)	0.0491^*a*^
Female, number (%)	38 (40.4%)	23 (57.5%)	0.0884^*b*^
White, number (%)	90 (97.8%)	39 (97.5%)	0.6694^*b*^
Laterality: right, number (%)	43 (45.3%)	23 (57.5%)	0.2580^*b*^
ESR (mm/h), median (IQR)	48.5 (39)	12.0 (16)	<0.001^*a*^
CRP (mm/dL), median (IQR)	45.5 (58.2)	7.0 (6.75)	<0.001^*a*^
Synovial fluid nucleated cell count (cells/mL), median (IQR)	38,950 (46,629)	1,862 (2,853)	<0.001^*a*^
Synovial fluid neutrophil percentage, median (IQR)	92 (10)	7 (23)	<0.001^*a*^

^*a*^ Wilcoxon rank-sum test.

^*b*^ Fisher’s exact test.

^*c*^ Erythrocyte sedimentation rate (ESR), C-reactive protein (CRP), inter-quartile range (IQR).

### Proteome of PJI highlights innate immunity

**Fig 1:**
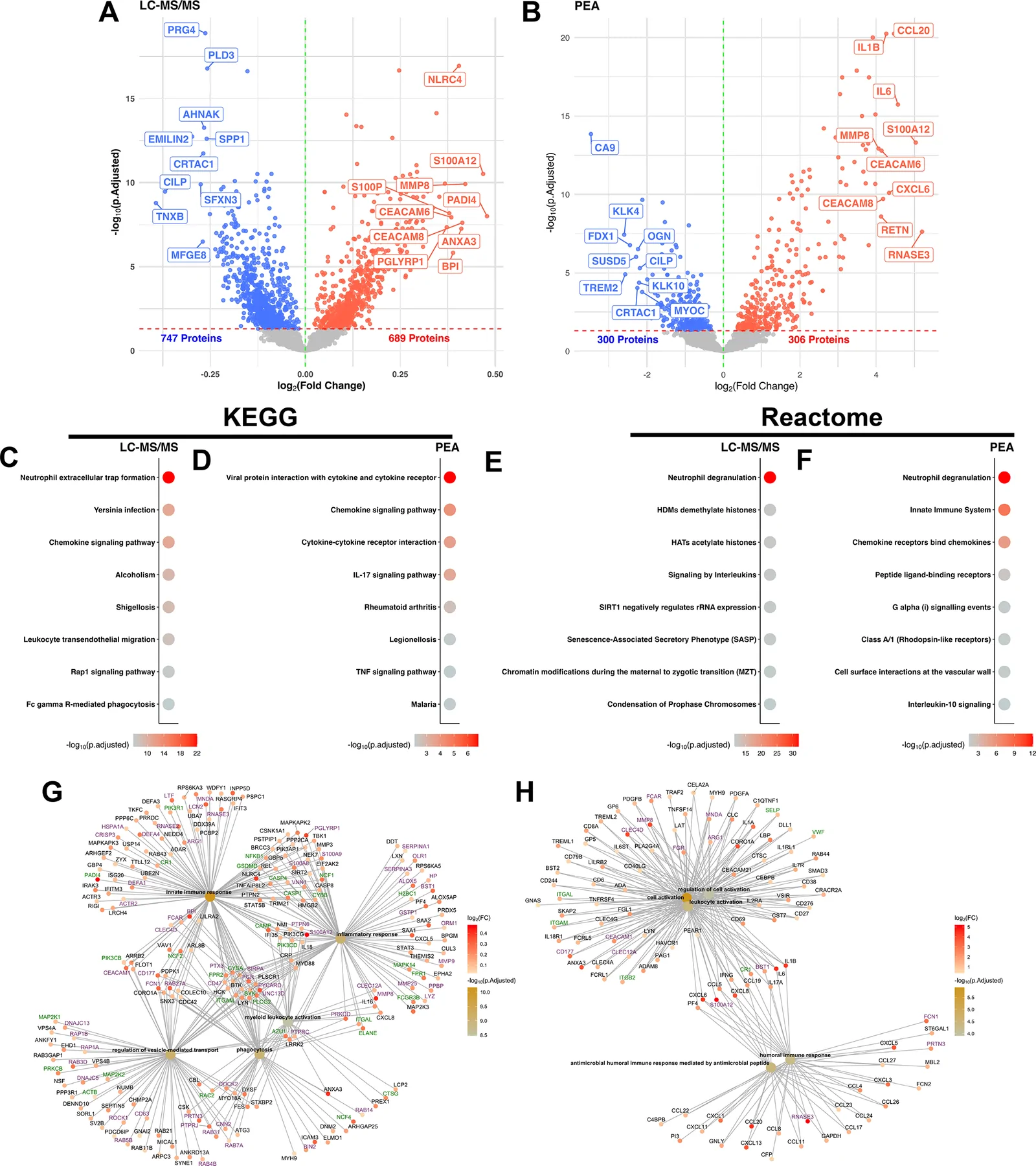
The host response to periprosthetic joint infection (PJI) is predominantly neutrophil-mediated. (**A and B**) The volcano plot demonstrates protein distribution based on fold change and adjusted *P-*values between non-infected arthroplasty failure and PJI for (**A**) liquid chromatography tandem mass-spectrometry (LC-MS/MS) and (**B**) proximity extension assay (PEA). The dashed lines represent thresholds used for gene enrichment analysis; red indicates an adjusted *P*-value < 0.05, and green log_2_FC < 0 or > 0. The 10 differentially abundant proteins with the largest fold change in each direction are labeled. Red and blue indicate an increase or decrease in PJI, respectively; gray indicates an adjusted *P*-value > 0.05. (**C and D**) Dot plots of the top 8 Kyoto Encyclopedia of Genes and Genomes (KEGG) pathways enriched in PJI from (**E**) LC-MS/MS and (**F**) PEA. (**E and F**) Dot plots of the top 8 Reactome pathways enriched in PJI by (**E**) LC-MS/MS and (**F**) PEA. (**G and H**) Gene-concept network map of the top five Gene Ontology (GO) terms enriched in PJI for (**G**) LC-MS/MS and (**H**) PEA. *P-*values for pathway enrichment were calculated through hypergeometric distribution; adjusted *P-*values were calculated through the Benjamini–Hochberg procedure.

To evaluate proteomic differences between PJI and NIAF, univariate analysis was performed on the 5,793 proteins identified by LC-MS/MS and the 2,888 proteins identified by PEA. LC-MS/MS and PEA revealed 1,436 (24.8%) and 600 (21.0%) DAPs between PJI and NIAF, respectively (Fig. 1A and B; Tables S8 and S9). Of the DAPs, LC-MS/MS and PEA revealed 689 and 306 proteins increased in abundance in PJI, respectively, with 87 proteins concordantly increased between methods (Fig. S1A). There was a strong correlation in the signed significance scores [defined as the −log_10_(adjusted *P*-value) × sign(log_2_ fold change)] for all DAPs identified by both LC-MS/MS and PEA (Spearman ρ = 0.805, *P*-value < 0.001) (Fig. S1B). Together, these data suggest that LC-MS/MS and PEA are complementary platforms that yield concordant results for shared targets and, when used together, enhance the depth of proteomic characterization between PJI and NIAF.

Across LC-MS/MS and PEA, DAPs with the greatest fold changes increased in PJI were largely associated with neutrophils and other innate immune processes (Table 2). Nine of 10 most increased proteins identified by LC-MS/MS were either primarily produced by neutrophils or related to neutrophil effector mechanisms, including peptidyl arginine deiminase 4 (PADI4), S100 calcium-binding protein A12 (S100A12), matrix metallopeptidase 8 (MMP8), carcinoembryonic antigen-related cell adhesion molecule 8 (CEACAM8), annexin A3 (ANXA3), NLR family CARD domain-containing protein 4 (NLRC4), bactericidal/permeability-increasing protein (BPI), carcinoembryonic antigen-related cell adhesion molecule 6 (CEACAM6), and peptidoglycan recognition protein 1 (PGLYRP1). Four of these (S100A12, MMP8, CEACAM6, CEACAM8) were increased with PEA. The remaining proteins increased in PJI from the PEA data set were related to neutrophil recruitment, activation, or function, including ribonuclease A family member 3 (RNASE3), interleukin-6 (IL-6), C-C motif chemokine ligand 20 (CCL20), C-X-C motif chemokine ligand 6 (CXCL6), interleukin-1-β (IL-1β), and resistin (RETN). Collectively, increased proteins in PJI across the LC-MS/MS and PEA data sets point to a host response skewed toward recruitment and activation of neutrophils.

**TABLE 2:** Top 10 differentially abundant proteins in periprosthetic joint infection measured by liquid chromatography tandem mass-spectrometry and proximity extension assay

Uniprot	Protein name	Gene symbol	Log_2_ fold change	Adjusted *P-*value
Liquid chromatography tandem mass spectrometry (LC-MS/MS)				
Q9UM07	Protein-arginine deiminase type-4	PADI4	0.478	9.96E-09
P80511	Protein S100-A12	S100A12	0.468	3.04E-11
P22894	Neutrophil collagenase	MMP8	0.421	1.21E-10
P31997	Cell adhesion molecule CEACAM8	CEACAM8	0.413	2.43E-08
P12429	Annexin A3	ANXA3	0.411	5.49E-08
Q9NPP4	NLR family CARD domain-containing protein 4	NLRC4	0.404	1.13E-17
P17213	Bactericidal permeability-increasing protein	BPI	0.389	1.50E-06
P40199	Cell adhesion molecule CEACAM6	CEACAM6	0.385	1.60E-08
P25815	Protein S100-P	S100P	0.379	6.71E-09
O75594	Peptidoglycan recognition protein 1	PGLYRP1	0.372	4.51E-08
Proximity extension assay (PEA)				
P12724	Eosinophil cationic protein	RNASE3	5.198	2.41E-08
P80511	Protein S100-A12	S100A12	5.033	4.93E-14
P05231	Interleukin-6	IL6	4.567	1.86E-16
P78556	C-C motif chemokine 20	CCL20	4.465	5.62E-21
P80162	C-X-C motif chemokine 6	CXCL6	4.335	8.01E-11
P01584	Interleukin-1 beta	IL1B	4.261	5.65E-21
P31997	Cell adhesion molecule CEACAM8	CEACAM8	4.180	1.94E-10
P40199	Cell adhesion molecule CEACAM6	CEACAM6	4.139	1.63E-13
Q9HD89	Resistin	RETN	4.126	2.59E-09
P22894	Neutrophil collagenase	MMP8	4.058	1.20E-13

Mapping DAPs to functional pathways offers insights into overrepresented mechanisms prominent in proteomics data sets (57). To understand the host response in PJI inferred from the sonicate fluid proteome, overrepresentation analysis (ORA) was performed on significantly increased and decreased DAPs. LC-MS/MS identified multiple KEGG pathways of translation and metabolism that were downregulated in PJI, including “ribosome,” “citrate cycle (TCA),” and “carbon metabolism” (Fig. S1C). In contrast, upregulated KEGG pathways identified via LC-MS/MS were enriched for immune and bacterial infection pathways, most notably with “NET formation” and “chemokine signaling pathway” (Fig. 1C). Similarly, PEA demonstrated enrichment of immune and bacterial infection subcategories, along with signaling molecule pathways, such as “viral protein interaction with cytokine and cytokine receptor” (Fig. 1D). No pathways were enriched for the decreased DAPs identified by PEA. Cell marker enrichment analysis with both LC-MS/MS and PEA detected increased myeloid cell populations, with neutrophils being the most significantly enriched in PJI, and a shared decrease in diverse structural cell signatures (Fig. S1E through H). Reactome pathway analysis further corroborated this finding, with “neutrophil degranulation” being the top enriched pathway by both LC-MS/MS and PEA (Fig. 1E and F). Gene Ontology ORA highlighted innate immune processes in LC-MS/MS and humoral responses in PEA, both incorporating proteins from NET formation and neutrophil degranulation (Fig. 1G and H). To determine if this neutrophil proteomic profile was shared across different etiologies, microorganism-specific PJIs were compared with NIAF. The four most prevalent causal pathogens in the cohort exhibited highly conserved host protein abundance profiles, with strong pairwise Spearman correlations ranging from 0.70 to 0.89 across both platforms (Fig. S1I and J). Overall, these findings suggest the host response in PJI is defined by neutrophil effector mechanisms, specifically NET formation and neutrophil degranulation.

### Neutrophil-related proteins discriminate PJI from NIAF

**Fig 2:**
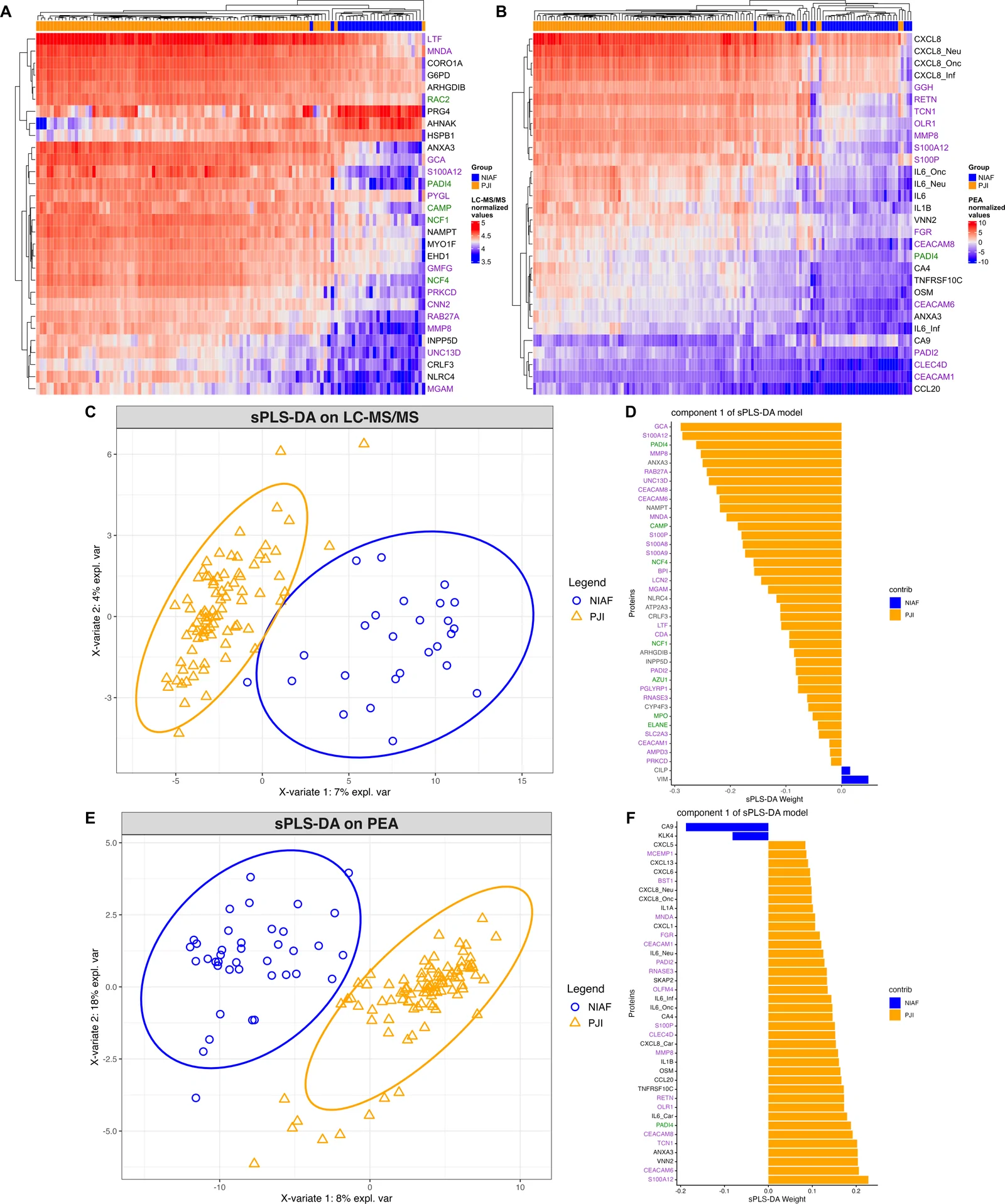
Cutpoint and multivariate analyses identify neutrophil extracellular trap formation and degranulation proteins among major discriminators of periprosthetic joint infection (PJI) from non-infectious arthroplasty failure (NIAF). (**A and B**) Heatmap analysis of (**A**) liquid chromatography tandem mass-spectrometry (LC-MS/MS) and (**B**) proximity extension assay (PEA) normalized values in PJI and NIAF, with the top 30 predictors identified from the optimal cutpoint analysis clustered using hierarchical clustering (Euclidean distance, complete linkage). (**C and E**) Sparse partial least squares discriminant analysis (sPLS-DA) demonstrating separation of sonicate fluid samples from PJI and NIAF by (**C**) LC-MS/MS and (**E**) PEA. (**D**) Mean maximum contribution score from component 1 of LC-MS/MS sPLS-DA model. (**F**) Mean maximum contribution score from top forty features of component 1 of PEA sPLS-DA model. Gene symbols in green belong to the Kyoto Encyclopedia of Genes and Genomes (KEGG) “neutrophil extracellular trap formation” pathway; gene symbols in purple belong to the Reactome “neutrophil degranulation” pathway. Ellipses represent the 95% confidence interval.

Univariate analysis and functional pathway analysis identified innate immune response proteins as increased in PJI. These findings prompted further univariate analysis to cross-validate neutrophil predominant proteins to strengthen robustness of observations. To evaluate the ability of individual proteins from the sonicate fluid proteome to distinguish PJI from NIAF, the cutpointr package was used to generate AUC values from receiver operating curves. The top 30 proteins from the LC-MS/MS and PEA data sets, based on AUC values, were used to perform hierarchical clustering of PJI and NIAF proteomes, which were visualized using heatmaps. In the LC-MS/MS data set, 83 of 85 of PJI samples (97.6%) clustered together, while 25 of 26 NIAF samples (96.2%) also clustered together (Fig. 2A). Proteins from NET formation and neutrophil degranulation predominately drove separation for LC-MS/MS, suggesting neutrophil effector mechanisms as strong discriminators between PJI and NIAF. In the PEA data set, 91 of 95 PJI samples (95.8%) and 32 of 39 NIAF samples (82.1%) clustered together, although separation was less distinct than by LC-MS/MS (Fig. 2B). In addition to NET and neutrophil degranulation proteins, IL-8 and IL-6 were prominently represented as proteins differentiating PJI from NIAF. These findings highlight a prominent role for IL-8, a chemoattractant for neutrophils, and IL-6, a mediator of the acute innate immune response, in the host immune response to PJI.

### Multivariate analysis underscores discriminatory potential of neutrophil proteins

Multivariate analysis is a complement to univariate analysis that captures collaborative effects and complex relationships within omics data sets (58). To holistically identify signatures to differentiate PJI from NIAF, the mixOmics package was used to sPLS-DA. Using LC-MS/MS data, the sPLS-DA model distinguished PJI from NIAF, largely driven by component 1 (Fig. 2C). Five-fold cross-validation yielded a mean AUC of 0.977. Features of component 2 and their respective weights revealed predominant contributions from proteins involved in NET formation and neutrophil degranulation (Fig. 2D). Similarly, using the PEA data, the sPLS-DA model separated PJI from NIAF, again driven largely by component 1 (Fig. 2E). Five-fold cross-validation yielded a mean AUC of 0.981 for the PEA model. Along with neutrophil effector mechanism pathways, IL-8 and IL-6—shared analytes represented on multiple Olink Explore panels—contributed to the top 40 features of component 1 (Fig. 2F). Overall, these findings recapitulate results from univariate analysis, emphasizing neutrophil effector mechanisms, IL-8, and IL-6, as prominent differentiators of PJI from NIAF.

### Upstream mechanisms of NET formation

The top KEGG pathway enriched based on LC-MS/MS was “NET formation” (Fig. 1D). Proteins within the pathway were repeatedly highlighted across analyses, including GO enrichment (Fig. 1H and I), univariate analysis with cutpointr (Fig. 2A and B), and driving features in the sPLS-DA model (Fig. 2D and F). To infer upstream mechanisms of NETosis enriched in PJI, the Pathview package was used to map elevated proteins in PJI onto the KEGG NET formation pathway. Enriched upstream mechanisms enriched in both data sets included “complement and coagulation cascades” and “platelet activation” (Fig. S2A). Notably, Siglec-9, which, upon binding to sialic acids or hyaluronic acid, dampens neutrophil activation and NETosis (59–62), was increased in abundance across both data sets. These findings suggest potential stimuli for NETosis in PJI, as well as a potential role of hyaluronate in synovial fluid in negatively modulating neutrophil activity.

### Synovial and sonicate fluid share a similar proteome

Sonicate fluid, analyzed here, is collected from resected implant surfaces (31, 32); synovial fluid is another fluid specimen collected from failed arthroplasties. To explore whether proteomic shifts associated with infection are consistent across these two joint-derived fluids, univariate and multivariate analyses were performed on a published synovial fluid data set of PJI and NIAF (50). Univariate analysis failed to reveal significant DAPs, with *P*-values adjusted for multiple comparisons testing all >0.05 (Fig. S3A). Based on the analysis done in reference 50, DAPs with unadjusted *P*-values < 0.05 were used in ORA. Top KEGG and Reactome pathways enriched among DAPs increased in PJI included “neutrophil extracellular trap formation,” “IL-17 signaling pathway,” “neutrophil degranulation,” and “innate immune system” (Fig. S3B and C). This suggests that the underlying host proteomic signatures in synovial fluid are consistent with those identified in sonicate fluid.

To assess possible protein differentiators of PJI shared across sonicate and synovial fluid, the two data sets were merged using the shared 456 proteins and used for sPLS-DA modeling. Separation of PJI from NIAF was largely driven by component 1, with no clear distinction between fluid types (Fig. S3D). Five-fold cross-validation calculated a mean AUC of 0.945 for the sPLS-DA model. Features in component 1 had proteins from both neutrophil degranulation and NET formation pathways (Fig. S3E). Finally, shared DAPs from sonicate and synovial fluid, measured by LC-MS/MS, correlated between their signed significance scores (defined as the −log_10_(*P*-value) × sign(fold change)) (Spearman ρ = 0.788, *P*-value < 0.001) (Fig. S3F). Overall, this suggests that the synovial fluid proteome mirrors trends in sonicate fluid and that NETs are differentially abundant in both specimen types.

### Artificial synovial fluid modulates NET formation

Here, NET formation in an *in vitro* environment mimicking features of a periprosthetic joint space was modeled. Given that PJI pathogens establish biofilms on prosthetic surfaces (5, 63), NETosis was studied in the context of biofilms. Neutrophils were dyed with cell-permeable and cell-impermeable nuclear stains to distinguish live cells from those with expelled nucleic material, the latter inferred as NETosis. Biofilms on glass beads (8.75 × 10^4^ CFU *S*. *aureus* IDRL-12882 [Fig. S4A]) and the sterilized supernatant from biofilm-grown cells were tested for their ability to induce NETs. Biofilms on beads induced and subsequently degraded NETs more rapidly than did filter-sterilized supernatant (Fig. S4B). This suggests that while secreted cell factors in the biofilm supernatant can induce NETosis, surface-bound biofilms may be preferred to study complex dynamics of NET formation in the context of *S. aureus* PJI. For this reason, the model further studied incorporated bead biofilms rather than relying on supernatants.

**Fig 3:**
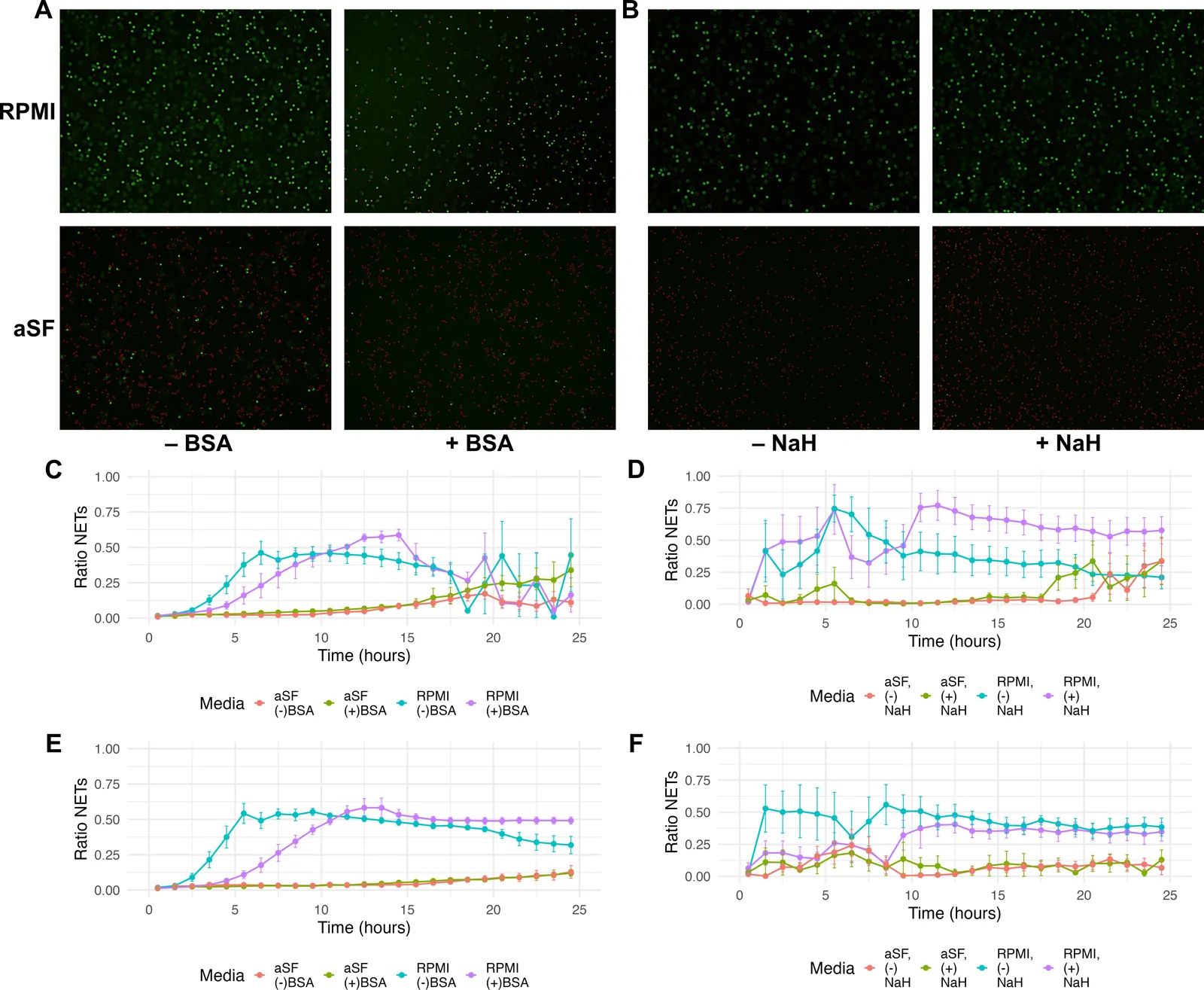
Impact of synovial fluid components on neutrophil extracellular trap (NET) formation using an *in vitro* biofilm NETosis model. (**A and B**) Representative images of healthy human neutrophils exposed to *Staphylococcus aureus* IDRL-12882 biofilms for 16.5 h in Roswell Park Memorial Institute (RPMI) or artificial synovial fluid (aSF) media, supplemented with or without (**A**) bovine serum albumin (BSA) or (**B**) sodium hyaluronate (NaH). Neutrophils were stained with membrane-impermeable Sytox Green and membrane-permeable NUCLEAR-ID red dye prior to time-lapse imaging. (**C and D**) Quantification of time lapse images of NETs induced by (**C**) *S. aureus* biofilms or (**D**) sterile beads in RPMI or aSF media, supplemented with or without BSA. (**E and F**) Quantification of time lapse images of NETs induced by (**E**) *S. aureus* biofilms or (**F**) sterile beads in RPMI or aSF media, supplemented with or without NaH. The ratio of NETs was calculated by dividing numbers of NETs imaged by the total cell numbers (live neutrophils plus NETs).

To examine dynamics of NET formation in synovial fluid, human neutrophils were stimulated with *S. aureus* IDRL-12882 bead biofilms or sterile controls in RPMI or aSF. Representative images from a single healthy subject’s neutrophils stimulated by *S. aureus* biofilms in RPMI or aSF media, with or without supplementation with BSA or sodium hyaluronate, are shown in Fig. 3A and B, respectively. Live cells were stained using a cell-impermeable DNA stain (red), while cell-permeable DNA stain-based quantification of extracellular neutrophil DNA was used as a proxy for NET formation (green). In RPMI, percent NET formation peaked at 3.0 and 4.5 h from *S. aureus* biofilm-induced and sterile beads, respectively (Fig. S4C). Interestingly, NET formation in aSF was delayed, suggesting potential components delaying or inhibiting initiation of NET formation.

BSA and hyaluronic acid inhibit formation of NETs in RPMI (60, 64). Albumin and hyaluronate are abundant in human synovial fluid (65–67); therefore, it was hypothesized that these components could have the same inhibitory effects in aSF. To test the impact of BSA on formation of NETs in aSF in comparison to RPMI, *S. aureus* biofilm-induced NET formation was quantified in aSF and RPMI in the presence and absence of BSA or hyaluronic acid. Addition of BSA to RPMI delayed NET formation at the 2.5–7.5 h time points (adjusted *P*-value < 0.05) compared with RPMI alone (Fig. 3C). In contrast, there was no inhibitory effect of BSA in aSF, with a trend toward a possible effect at later time points (Fig. 3C). These findings replicate previous findings of the inhibitory effects of BSA on NET formation in RPMI (64), but do not demonstrate similar inhibitory effects in aSF, which may be more reflective of the joint environment. Hyaluronic acid demonstrated no effect on NETs under *S. aureus* induction in either RPMI or aSF (Fig. 3E). Under sterile bead conditions, sodium hyaluronate delayed NET formation in RPMI, whereas no effect was observed in aSF (Fig. 3F). Overall, these findings indicate that synovial fluid components other than BSA and hyaluronic acid may have immunomodulatory effects on neutrophil effector mechanisms, leading to delayed NET formation compared to that observed *in vitro* in RPMI.

## DISCUSSION

In this study, the physiology of NETs in PJI was investigated using human proteomics and advanced *in vitro* time-lapse microscopy with custom image analysis. First, complementary LC-MS/MS and PEA approaches were used to profile the host proteome of sonicate fluid from patients of PJI and NIAF to understand the immune response in PJI. These methods identified 908 unique proteins increased in abundance in PJI, with 87 shared between LC-MS/MS and PEA. Significantly increased DAPs in PJI were enriched for innate immune responses, including NET formation and neutrophil degranulation, highlighting neutrophils as a prominent mediator in PJI. An *in vitro* PJI NET model was developed to study effects of synovial fluid components on NET formation. NET formation was delayed in aSF compared to RPMI, indicating the importance of studying NETosis using physiologically relevant fluid, rather than in cell culture media. Together, these findings highlight NET formation as a key feature of PJI and establish an experimental platform for further study of PJI NETs.

PEA technology, while limited to detection of proteins included in the panel, enables detection of low abundance proteins that may not be detected by LC-MS/MS as a result of masking by high abundance proteins. Thus, the high sensitivity of PEA approach is excellent for quantifying short-lived and low abundance cytokines in human samples (34, 68–70). In contrast, LC-MS/MS, which is not limited by antibodies, detects a broader range of proteins. Here, LC-MS/MS samples were run on concentrated sonicate fluid samples, while PEA was run on neat samples. Given methodological and sample differences, it is not surprising that there were some differences across the two platforms, including some differences in DAPs identified by each. Despite this, LC-MS/MS and PEA platforms showed concordance across shared DAPs and pointed to similar ORA pathways.

Neutrophils play a central role in the innate immune response, and their presence in synovial fluid and periprosthetic tissue is a marker of PJI (71, 72). Consistent with this, PEA revealed increased abundance of neutrophil-recruiting chemokines (CXCL1-CXCL8), especially CXCL8 (IL-8), and pro-inflammatory markers, such as IL-6, IL-1α, IL-1β, and IL-17A. This aligns with increased neutrophil-associated DAPs in PJI found by LC-MS/MS, including S100A8, S100A9, S100A12, MPO, ELANE, lactoferrin, AZU1, BPI, and PADI4. These findings from human samples are consistent with findings from a murine tibial implant model, where mice with implants infected with *S. aureus* showed increased expression of IL-1β, IL-6, CXCL1, and CXCL3 genes compared to non-infected controls (73). Here, functional analysis of DAPs from both analyses demonstrated that NET formation and neutrophil degranulation pathways were enriched in PJI; prominent proteins from each pathway were further highlighted by multivariate analysis. These findings are concordant with investigations identifying byproducts of NET formation as significantly upregulated in synovial fluid (18, 74) and plasma (20), underscoring the diagnostic potential of NETs. Some have suggested that NET formation could play a pathogenic role in aseptic arthroplasty failure (75, 76). While NET formation was shown here to be downregulated in NIAF relative to PJI, it is possible that NETs are sufficiently abundant in NIAF to have a pathogenic role. Furthermore, the robust NETosis signature observed in PJI suggests the presence of dysregulated neutrophil effector mechanisms as being important in disease pathogenesis, warranting further exploration as to how aberrant NET formation may drive pathology.

Although NET-associated proteins were elevated in PJI, the synovial joint space represents a distinct immune microenvironment that may shape NET production, stability, or clearance. Further, microorganisms causing PJI have demonstrated a variety of NET evasion tactics, including enzymatic degradation of DNA scaffolds and the co-option of extracellular DNA to improve biofilm stability (77–80). Previous work showed that albumin and hyaluronic acid—both abundantly present in synovial fluid and aSF—inhibit NET formation *in vitro* (60, 64, 81). Data presented here demonstrate a decrease in NETosis in aSF compared to RPMI. This study sought to perform *in vitro* modeling of NET formation induced by biofilms in the synovial fluid microenvironment, specifically profiling the effects of albumin and hyaluronic acid. While albumin delayed *S. aureus* biofilm-induced NETs when added to RPMI, albumin and hyaluronic acid did not inhibit NETosis at 24 h when added to aSF. Adding sodium hyaluronic acid to RPMI or aSF only delayed NET formation under sterile conditions and appeared to have no effect on *S. aureus* biofilm-induced NETs. These findings suggest that aSF components other than albumin or hyaluronic acid may be responsible for inhibiting or delaying NETosis. That, or factors other than bacterial stimulation or joint nutritional components missing from the model—such as host cytokines and local tissue niches—are responsible for driving NETosis. Notably, NETosis induced by *S. aureus* biofilms did not surpass NET levels from sterile beads. While the model incorporates a synovial fluid substitute, it lacks immune cells and cytokines that promote NET production in the presence of pathogens. Additionally, *S. aureus* employs multiple mechanisms that dysregulate the bactericidal potential of NETosis (80, 82), including degradation of DNA that represents the backbone of NETs (83, 84). Together, these results suggest that, in addition to being stimulated by biofilms, the dynamics of NET formation are impacted by other unknown factors in the joint environment, such as cytokine gradients, cellular composition, and/or local tissue niches; further studies are needed to determine why NETs are ineffective in clearing PJI.

There are several limitations to this study. First, the use of proteomics may introduce bias in enrichment analysis, as the type of “omics” platform inherently shapes the detectable molecular landscape. To address this limitation, rigorous statistical methods were implemented, including multiple comparisons adjustment for differential abundance and ORA, background gene sets for ORA, and transparency in the R package versions and databases used. Multiple statistical methods were implemented to enhance confidence in highlighted proteins. These measures collectively reduce the risk of false positive results and increase robustness. Second, this study utilized sonicate fluid derived from explanted prosthetics, which requires implant removal. Variability in retained tissue types and amounts across prostheses may introduce heterogeneity in protein recovery; standardized sonication and implant retrieval protocols and the large sample size help mitigate this risk. Third, quantification of *in vitro* NETs relied solely on intracellular and extracellular DNA staining, without incorporating other NET-associated protein markers, such as MPO or citrullinated H3-histones. While this technique captures nuclear material extrusion, it may not fully distinguish NET formation from other forms of cell death. This limitation is particularly consequential for the conclusion that artificial synovial fluid delays NET formation. Furthermore, sterile beads released extracellular DNA, possibly as a result of mechanical stimulation rather than pathogen-specific induction of NET formation. To mitigate this, the custom image analysis pipeline incorporated fluorescence intensity and morphometric parameters to enhance specificity for NETs. Fourth, although the clinical PJI cohort included multiple bacterial species, *in vitro* mechanistic experiments focused on *S. aureus*. While host proteomic signatures were shared among gram-positive organisms with adequate sample sizes (*S. aureus*, *S. epidermidis*, *E. faecalis*, and *S. lugdunensis*), the number of gram-negative PJIs was insufficient for comparable subgroup analysis. Because distinct pathogens express distinct pathogen-associated molecular patterns that may elicit differential neutrophil responses (85), these findings may not extend to gram-negative infections, which should be evaluated in future studies. Although the present study focused on the host response, future work should also evaluate whether specific bacterial factors, such as adhesins and biofilm-associated proteins, differentially influence NET dynamics within the synovial environment. Fifth, the *in vitro* model only involved bacteria and neutrophils and does not recapitulate the effects of cytokines or other immune cell populations. In particular, monocytes and macrophages are important contributors to the immune microenvironment in PJI and may influence NET formation, persistence, or clearance, alongside interactions with biofilms. This may partly explain why NETosis was delayed in aSF compared to RPMI. Future studies could employ cytokines and other immune factors, such as platelets, monocytes, and macrophages, to further investigate the dynamics of NETosis in PJI. Finally, a common limitation in PJI studies, also observed here, is the lack of racial diversity.

Despite efforts to mitigate the risk of developing PJI, PJI rates have remained relatively stable over the past decade. Further, current treatment for most PJIs requires prolonged antimicrobial therapy in combination with surgical intervention, representing a significant burden to patients and the healthcare system. Here, characterization of the host proteome in PJI compared to NIAF revealed an immune response in PJI dominated by the neutrophil effector mechanisms. When NETosis was modeled in a synovial fluid-like environment, its onset was delayed, underscoring the importance of modeling biologically relevant conditions to accurately capture host-pathogen interactions. Future studies should explore the molecular mechanisms regulating neutrophil effector function in synovial fluid and assess whether targeting these pathways could offer novel therapeutic strategies for PJI.

## Data Availability

The mass spectrometry proteomics data have been deposited to the ProteomeXchange Consortium via the PRIDE partner repository with the data set identifiers PXD080672 and PXD058084. Code is available at https://github.com/RPatelLab/NETs_Metaproteomics.

## SUPPLEMENTAL MATERIAL

Supplemental figures spectrum.00665-26-s0001.docx Fig. S1 to S4.

Supplemental tables spectrum.00665-26-s0002.xlsx Tables S1 to S10.

ASM does not own the copyrights to Supplemental Material that may be linked to, or accessed through, an article. The authors have granted ASM a non-exclusive, world-wide license to publish the Supplemental Material files. Please contact the corresponding author directly for reuse.
