# Supplementary material for: Metaproteomic analysis of human sonicate fluid highlights neutrophil extracellular trap formation associated with periprosthetic joint infection
Source: Microbiol Spectr. 2026 Oct 13;14(11):e00665-26. doi: 10.1128/spectrum.00665-26 (PMC13729098; doi:10.1128/spectrum.00665-26)
Supplement: Supplementary file 1 [file spectrum.00665-26-s0001.docx]

**Figure S1. Integrated proteomic analyses reveal concordance across platforms and pathogen-specific host protein signatures in periprosthestic joint infection (PJI).** (A) Upset plot showing interaction sizes between increased and decreased differentially abundant proteins (DAPs) between liquid chromatography tandem mass-spectrometry (LC-MS/MS) and proximity extension assay (PEA). (B) Spearman correlation of signed significance scores for all the proteins shared among LC-MS/MS and PEA. The dashed line represents a slope of 1. Signed significance scores were calculated by –log_10_(Adjusted *p-value*) * sign(Fold Change). (C, D) Dot plots of the top 8 (C) KEGG and (D) Reactome pathways enriched for decreased abundant proteins from LC-MS/MS. (E, F) Dot plots of the top 8 enriched cell types based on increased differentially abundant proteins by (E) LC-MS/MS and (F) PEA. (G, H) Dot plots of the top 8 enriched cell types based on decreased DAPs in (G) LC-MS/MS and (H) PEA. (I, J) Matrix of Spearman correlation coefficients (ρ) based on the signed significance scores for human protein signatures found in *Staphylococcus aureus*, *Staphylococcus epidermidis*, *Staphylococcus lugdunensis*, or *Enterococcus faecalis* PJI compared to non-infectious arthroplasty failure, measured by (I) LC-MS/MS and (J) PEA. *p-values* for pathway enrichment were calculated through hypergeometric distribution; adjusted *p-values* were calculated through the Benjamini-Hochberg procedure.

**Figure S2. Upstream mechanisms of neutrophil extracellular trap formation shared between liquid chromatography tandem mass-spectrometry (LC-MS/MS) and proximity extension assay (PEA).** Pathview analysis of the Kyoto Encyclopedia of Genes and Genomes (KEGG) pathway neutrophil extracellular trap formation (“hsa04613”) for LC-MS/MS and PEA data. The color scale represents scaled fold changes between periprosthetic joint infection (PJI) and non-infectious arthroplasty failure (NIAF): red and blue represent increased and decreased abundance in PJI, respectively; gray indicates that the protein was either not measured or not significantly differentially abundant.

**Figure S3. Comparison of synovial and sonicate fluid host proteomes in periprosthetic joint infection (PJI).** (A) Volcano plot demonstrating the distribution of proteins in synovial fluid based on fold change and adjusted p-values between non-infectious arthroplasty failure (NIAF) and PJI. Dashed lines represent thresholds used in gene enrichment analysis; black indicates a *p-value* <0.05, red an adjusted *p-value* <0.05, and green a log_2_FC <0 or >0. Red indicates an increase in PJI, blue a decrease in PJI, and grey a *p-value* >0.05. (B, C) Dot plot of the top 8 (B) Kyoto Encyclopedia of Genes and Genomes (KEGG), and (C) Reactome pathways enriched by over-representation analysis for increased differentially abundant proteins in PJI. (D) Sparse partial least squares discriminant analysis (sPLS-DA) demonstrating separation of sonicate and synovial fluid samples from PJI and NIAF from liquid chromatography tandem mass-spectrometry (LC-MS/MS). (E) Mean maximum contribution score from components 1 and 2 in the sonicate and synovial fluid sPLS-DA model. (F) Spearman correlation of signed significance scores for differentially abundant proteins shared among LC-MS/MS synovial and sonicate fluid samples. Signed significance scores were calculated by -log10(Adjusted *p-value*) * sign(Fold Change). Gene symbols in green belong to the KEGG “neutrophil extracellular trap formation” pathway; gene symbols in purple belong to the Reactome “neutrophil degranulation” pathway*.* *p-values* for pathway enrichment were calculated through hypergeometric distribution, and adjusted *p-values* were calculated through the Benjamini-Hochberg procedure.

**Figure S4. Effects of *in vitro Staphylococcus aureus* biofilms on neutrophil extracellular trap formation.** (A) Quantification of colony forming units (CFU) adherent to bead biofilms; n = 5. (B) Ratio of neutrophil extracellular traps (NETs) induced by *S. aureus* IDRL-12882 bead biofilms compared to sterilized biofilm supernatant over time; n = 2. (C) Ratio of NETs induced by *S. aureus* IDRL-12882 in artificial synovial fluid (aSF) compared to Roswell Park Memorial Institute Medium (RPMI) media over time; n = 7. Ratio of NETs was calculated by dividing numbers of NETs imaged by total cell numbers (live neutrophils plus NETs). Limit of detection (LOD).
